# Hybrid Curriculum Learning for Data-Efficient Lung Nodule Detection with YOLOv11

**DOI:** 10.3390/diagnostics16101441

**Published:** 2026-05-08

**Authors:** Yi Luo, Yike Guo, Hamed Hooshangnejad, Xue Feng, Quan Chen, Zongwei Zhou, Yaxi Chen, Yipeng Hu, Rui Zhang, Kai Ding

**Affiliations:** 1 Department of Biomedical Engineering, Johns Hopkins University, Baltimore, MD 21218, USA; yluo62@jh.edu (Y.L.); yguo122@jh.edu (Y.G.); 2 Department of Radiation Oncology and Molecular Radiation Sciences, Johns Hopkins University, Baltimore, MD 21287, USA; h.hooshangnejad@gmail.com; 3 Carina Medical LLC, Lexington, KY 40513, USA; xfeng@carinaai.com; 4 Department of Radiation Oncology, Mayo Clinic Arizona, Phoenix, AZ 85054, USA; chen.quan@mayo.edu; 5 Department of Computer Science, Johns Hopkins University, Baltimore, MD 21218, USA; zzhou82@jh.edu; 6 Department of Mechanical Engineering, University College London, London WC1E 7JE, UK; yaxi.chen.20@ucl.ac.uk; 7 UCL Hawkes Institute, University College London, London WC1E 6BT, UK; yipeng.hu@ucl.ac.uk; 8 Department of Medical Physics and Biomedical Engineering, University College London, London WC1E 6BT, UK; 9 Division of Computational Health Sciences, Department of Surgery, University of Minnesota, Minneapolis, MN 55455, USA

**Keywords:** lung nodule detection, YOLO, curriculum learning, chest CT, data scarcity

## Abstract

**Background/Objectives:** Accurate detection of pulmonary nodules on chest CT is critical for lung cancer screening, yet training robust detectors remains challenging due to the high cost of reliable annotations. In this work, we present a systematic study of curriculum learning for CT-based lung nodule detection on the enhanced LUNA25 benchmark and propose a hybrid curriculum learning framework for data-efficient optimization. **Methods:** Our approach estimates sample difficulty by fusing clinically interpretable handcrafted factors such as nodule size and count with model-driven signals such as prediction confidence from a teacher model, and constructs a three-stage progressive training curriculum from easy to hard samples. Using YOLOv11s as a strong baseline, the proposed hybrid curriculum is compared against conventional training without curriculum learning and against single-source curricula. **Results:** On the held-out LUNA25 test set, hybrid curriculum learning increases ${\mathrm{mAP}}_{50}$ from 0.672 to 0.696, ${\mathrm{mAP}}_{50–95}$ from 0.369 to 0.385, recall from 0.588 to 0.634, and precision from 0.725 to 0.764. Extensive data-efficiency experiments with proportional reductions (1/2, 1/5, 1/10) and fixed training samples (5000–40,000 slices) further confirm consistent gains across limited-data regimes. **Conclusions:** These results demonstrate that jointly leveraging intrinsic image complexity and optimization-aware feedback provides effective sample scheduling for robust and data-efficient lung nodule detection.

## 1. Introduction

Lung cancer remains a major global health burden and is one of the leading causes of cancer-related mortality worldwide [1]. In the United States, more than 238,000 new cases are diagnosed each year [2], while the overall five-year survival rate is still below 20% despite ongoing advances in diagnosis and treatment [3].

Early detection is therefore critical for improving treatment outcomes, as survival rates drop substantially with advancing disease stage [4]. Computed tomography (CT) is widely used for lung cancer screening due to its high spatial resolution and its ability to depict detailed pulmonary structures [5]. This enables the identification of subtle abnormalities, including small pulmonary nodules that may represent early-stage lung cancer [6]. Such nodules typically appear as round opacities or irregular lesions, making accurate detection and characterization challenging in clinical practice [7].

Recent advances in deep learning have significantly improved the performance of computer-aided detection (CAD) systems for pulmonary nodule analysis [8,9,10,11]. Nevertheless, most existing studies primarily focus on architectural design or feature enhancement, while training strategies typically adopt uniform sample scheduling during optimization [12,13,14,15,16]. In complex medical imaging datasets, where lesion appearance and detection difficulty vary substantially across samples, uniform training may limit model robustness and generalization.

Curriculum learning (CL) is a training paradigm inspired by human learning, in which training samples are presented according to an estimated difficulty score rather than uniform random shuffling [17]. Difficulty in CL can be estimated from different sources. Handcrafted signals are computed directly from the input data using predefined rules or domain knowledge, such as lesion size, image contrast, or annotation complexity. Detector-driven signals, in contrast, are derived from a trained model’s behavior on each sample, for example prediction confidence, loss magnitude, or uncertainty. By organizing training data by difficulty levels, CL can stabilize optimization and improve feature learning, particularly in scenarios with heterogeneous data distributions. Such difficulty-aware training strategies may be particularly well-suited for medical imaging, where lesions often exhibit large variability in scale, contrast, and structural complexity.

Despite its potential, existing curriculum learning studies do not fully address the challenges of CT-based lung nodule detection. Prior curriculum learning research in medical imaging has mainly focused on segmentation and classification tasks [18,19,20], while object detection introduces additional challenges such as localization ambiguity, scale variation, structures that are prone to false-positive detections, and image quality heterogeneity. As a result, formulating curriculum learning for robust lung nodule detection on heterogeneous CT data remains insufficiently explored.

**Figure 1:**
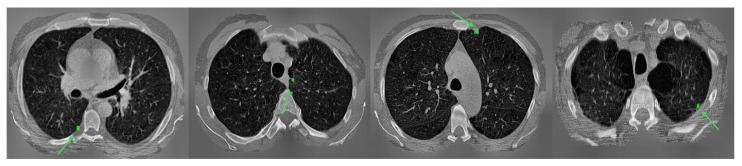
Representative challenging slices from the enhanced LUNA25 dataset. Green boxes indicate the nodule locations. The selected examples highlight small nodules located adjacent to surrounding anatomical structures, illustrating the increased detection difficulty caused by limited lesion size, reduced conspicuity, and interference from anatomical structures.

Most existing studies on lung nodule detection rely on the LUNA16 dataset [21], which long served as the main benchmark for model development and evaluation. In contrast, the original LUNA25 release was primarily designed for malignancy risk estimation rather than precise detection supervision [22]. The recently enhanced annotation version, generated through the foundation model MedSAM2 followed by expert refinement, provides refined pixel-level nodule delineations that make detection-oriented training and evaluation feasible [23]. Importantly, enhanced LUNA25 includes 4069 low-dose chest CT examinations and exhibits substantial variation in lesion size, lesion count, morphology, and slice quality. These characteristics make it a more diverse and heterogeneous benchmark for lung nodule detection, providing a valuable setting for assessing curriculum learning strategies at a larger scale. Figure 1 illustrates representative visually challenging cases from the enhanced LUNA25 dataset. In particular, the selected slices include small nodules and nodules adjacent to surrounding anatomical structures, highlighting the increased difficulty of detecting subtle lesions in anatomically complex regions.

To address this research gap, we present a systematic study of curriculum learning for CT-based lung nodule detection on the LUNA25 dataset. This work makes three main contributions. First, we establish benchmark performance for state-of-the-art YOLO detection models on LUNA25, providing a standardized reference for future studies. Second, we propose a hybrid curriculum learning framework tailored to CT-based lung nodule detection, which integrates clinically grounded handcrafted factors with specific model-driven signals. Third, we evaluate the proposed approach across multiple data scales, including cross-validation splits and reduced training subsets, to assess its robustness under the limited-data conditions common in medical imaging. Experiments on the LUNA25 dataset show that the proposed framework consistently improves detection performance over conventional training and over single-source curricula design, with particularly stable gains under reduced training budgets.

## 2. Related Work

Deep learning has significantly advanced automated lung nodule detection in CT imaging. Early studies mainly relied on two-stage detection frameworks, such as Faster R-CNN and Mask R-CNN adapted for medical imaging, where candidate regions were first generated and then refined through classification networks [24,25]. Although these approaches often achieve high detection sensitivity, their multi-stage pipelines typically introduce increased computational cost and slower inference speed. More recent work has increasingly shifted toward one-stage detectors, which jointly perform localization and classification within a unified framework to improve efficiency. Among these methods, YOLO-based architectures have gained growing attention due to their favorable balance between detection accuracy and computational efficiency, making them attractive for large-scale clinical imaging applications [26,27,28,29]. Despite these advances, most existing studies emphasize architectural improvements or feature enhancement, while training strategies are typically treated as fixed components during optimization.

CL has been extensively studied as a training strategy that provides performance improvements over the standard training approach based on random data shuffling [30]. Existing approaches can be broadly categorized according to how difficulty is estimated and how the curriculum is constructed during training. One major class of methods relies on predefined or externally defined curricula, where training samples are ranked using fixed criteria derived from prior knowledge, heuristic measures, or auxiliary models. Such strategies typically establish a static training schedule before optimization begins, providing interpretable and stable learning trajectories while reducing sensitivity to noisy or complex samples in early training stages [31,32]. In contrast, adaptive curriculum learning methods estimate sample difficulty dynamically based on model-dependent signals that change throughout optimization [33,34]. Representative formulations include self-paced learning, where sample selection is progressively expanded according to optimization-driven criteria, as well as approaches that rely on loss magnitude, prediction confidence, or uncertainty estimation to guide data sampling [35,36,37,38,39]. By continuously updating the curriculum according to the learner’s current state, adaptive strategies aim to align data presentation with model maturity, thereby improving training robustness and convergence behavior, particularly in heterogeneous or noisy datasets.

CL has recently been explored in various medical imaging applications to address data heterogeneity and optimization challenges. Existing studies mainly focus on tasks such as medical image segmentation and classification, where difficulty-aware sampling strategies have been shown to improve optimization stability and model performance [18,19,20]. These approaches typically leverage domain-specific priors or model-driven signals to guide the learning process and mitigate issues such as noisy annotations and scale variation. Despite these promising results, its application to object detection tasks is relatively limited. In particular, curriculum-based training strategies for CT-based lung nodule detection remain underexplored, motivating further investigation in this direction.

## 3. Method

### 3.1. Dataset

The LUNA25 grand challenge provides a large-scale, retrospective, multi-center benchmark consisting of 4069 low-dose chest CT examinations, curated to support the development and validation of modern artificial intelligence methods for lung nodule malignancy risk estimation, offering standardized imaging data and annotations that facilitate reproducible algorithmic evaluation. However, the original annotations were designed for malignancy assessment rather than detection, lacking precise nodule boundary delineations. We therefore adopted an enhanced annotation version based on MedSAM2 and expert refinement [23]. Slice-wise bounding box annotations were further derived from the pixel-level segmentation masks to enable detection-oriented training and evaluation.

### 3.2. Preprocessing

Our preprocessing pipeline consisted of four sequential stages designed to improve data quality and ensure reliable lung nodule detection model training: (1) lung segmentation using TotalSegmentator [40], followed by boundary expansion to guarantee complete anatomical coverage and avoid truncation of peripheral structures; (2) slice-wise image quality assessment was conducted based on lung coverage ratio, intensity heterogeneity, and structural conspicuity, allowing the exclusion of slices with limited informative content or suboptimal image quality; (3) a nodule-oriented slice sampling strategy was subsequently employed, in which all slices containing nodules were preserved, whereas only a subset of high-quality non-nodule slices was retained at an approximate 1:2 ratio to reduce data redundancy and achieve a more balanced training set; and (4) nodules measuring less than 3 mm in diameter were removed because of their limited clinical relevance. The remaining slices were then processed with contrast-limited adaptive histogram equalization (CLAHE) to enhance local contrast and facilitate the visualization of subtle pulmonary structures. All processed slices were resized to a unified resolution of 512 × 512, with aspect ratio preserved through padding when necessary to avoid geometric distortion. The resulting dataset consists of 58,999 high-quality CT slices collected from 4069 examinations, with slice-level bounding box annotations available for model development and evaluation. To ensure robust evaluation and prevent information leakage, patient-level data splitting was adopted so that all slices from a single patient were assigned exclusively to one subset, thereby reducing the risk of memorizing patient-specific imaging characteristics and improving generalization. The dataset was divided using an 80/10/10 patient-level split, such that all slices from the same patient were assigned exclusively to a single subset, thereby preventing overlap across the training, validation, and test sets. This resulted in 47,086 training slices from 3255 patients, 6357 validation slices from 406 patients, and 5556 test slices from 408 patients.

### 3.3. Hybrid Curriculum Learning Framework

**Figure 2:**
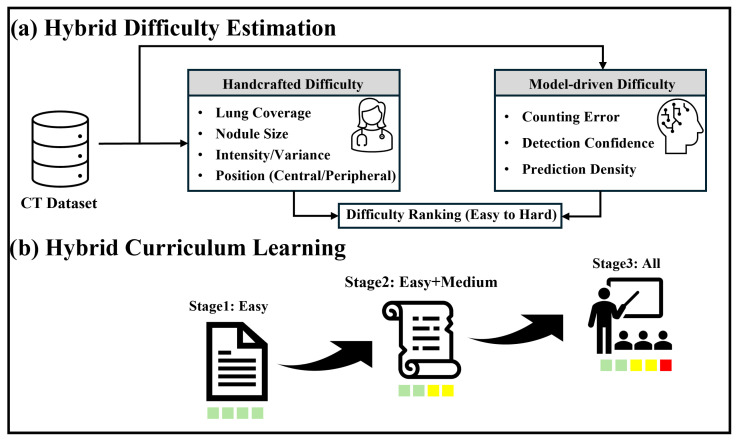
Overview of the proposed hybrid curriculum learning framework. (**a**) Hybrid difficulty estimation combining handcrafted and model-driven scores. (**b**) Progressive curriculum training schedule with increasing data complexity.

A key challenge in curriculum learning is the definition of sample difficulty, since the effectiveness of the curriculum depends on whether the assigned difficulty accurately reflects the learning complexity encountered by the detector. To capture both intrinsic image complexity and detector-dependent optimization behavior, we propose a hybrid curriculum learning framework that combines handcrafted and model-driven difficulty into a unified score. Figure 2 illustrates the overall structure of the proposed framework. As shown in Figure 2a, handcrafted difficulty and model-driven difficulty are computed in parallel from the CT dataset and fused into a unified hybrid score, which is used to rank samples from easy to hard. Based on this ranking, a progressive curriculum training schedule is constructed as shown in Figure 2b, where training proceeds through three stages with gradually increasing data complexity. The final curriculum difficulty is defined as a convex combination of handcrafted and model-driven scores,

(1) $$c_{\mathrm{hybrid}}=a\;c_{\mathrm{manual}}+\left(1-a\right)\;c_{\det},$$

where a∈[0,1] controls their relative contribution.

#### 3.3.1. Handcrafted Difficulty Definition

Each CT slice is assigned a handcrafted difficulty score computed as the sum of four clinically interpretable factors.

(2) $$c_{\mathrm{manual}}=f_{\mathrm{cnt}}+f_{\mathrm{size}}+f_{\mathrm{shape}}+f_{\mathrm{qual}}$$

These factors were designed to provide a coarse but interpretable ranking of sample difficulty based on intrinsic image characteristics relevant to lung nodule detection. The associated coefficients and thresholds were selected as heuristic, empirically chosen design rules, informed by preliminary inspection of the LUNA25 dataset. The four factors capture different aspects of detection difficulty. Specifically, $f_{\mathrm{cnt}}$ (nodule count) is assigned 0.5 for nodule-free slices, 1.0 for slices containing a single nodule, 2.5 for slices with 2–3 nodules, and 4.0 for slices with 4 or more nodules. The factor $f_{\mathrm{size}}$ (nodule size) is determined by the smallest nodule in the slice and is assigned 0.5 when no nodule is present, 1.0 when the minimum nodule area exceeds 1000 pixels, 2.0 for 400–1000 pixels, and 3.0 for smaller nodules. The factor $f_{\mathrm{shape}}$ (shape irregularity) is assigned 0.5 for regular nodules with low aspect-ratio variance, 1.0 when at most one irregular nodule is present, and 2.0 when multiple irregular nodules are observed, where irregularity is defined by extreme aspect ratios. Finally, $f_{\mathrm{qual}}$ (image quality) is assigned 0.5 for sharp, high-contrast images with Laplacian variance >500 and contrast >30, 1.0 for medium-quality images, and 2.0 for blurry or low-contrast images.

#### 3.3.2. Model-Driven Difficulty

To complement handcrafted priors with optimization-aware signals, we compute a model-driven difficulty score using a teacher detector. The dataset is split into 80% training and 20% held-out subsets; within the 80% training portion, 90% is used for training and 10% for validation to obtain the teacher model, which is then applied to the held-out subset. This splitting is independent of the cross-validation protocol used for performance evaluation and is solely intended for difficulty scoring. A held-out subset is used to ensure that difficulty scores are assigned on unseen samples, avoiding potential bias from in-sample scoring. For each slice, the model-driven difficulty is computed as

(3) $$c_{\det}=\min\left(\max\left(\lambda_{1}\left(1-\bar{p}\right)+\lambda_{2}\frac{|N_{\mathrm{pred}}-N_{\mathrm{gt}}|}{\max(N_{\mathrm{gt}},1)}+\lambda_{3}\min\left(0.5\;N_{\mathrm{pred}},\;2.0\right),\;0.5\right),\;8.0\right)$$

where $N_{\mathrm{pred}}$ is the number of predicted boxes, and $N_{\mathrm{gt}}$ is the ground-truth nodule count derived from annotations. $\bar{p}$ is defined as the mean confidence of post-NMS predicted boxes when $N_{\mathrm{pred}}\geq 1$, and as one minus the mean top-5 pre-NMS candidate score when $N_{\mathrm{pred}}=0$, so that higher $\bar{p}$ consistently indicates higher detector confidence. The three terms respectively penalize low-confidence predictions, counting discrepancies between predictions and ground truth, and overly dense predictions. In our implementation, we set $\lambda_{1}=\lambda_{2}=3.0$ and $\lambda_{3}=1.0$. These weights were selected as heuristic empirical settings to balance the relative contributions of prediction confidence, counting discrepancy, and prediction density in the curriculum ranking. Larger weights were assigned to the first two terms since low confidence and mismatch in predicted lesion count more directly reflect detector failure on a given sample, whereas the density term mainly serves as an auxiliary penalty for overly dense predictions and was therefore assigned a smaller weight. We further clip $c_{\det}$ to [0.5, 8.0] to prevent extreme outliers from dominating the curriculum ranking.

#### 3.3.3. Hybrid Curriculum Construction

Based on the hybrid difficulty scores, samples are categorized into simple ($c_{\mathrm{hybrid}}\leq 3$), medium ($3<c_{\mathrm{hybrid}}\leq 6$), and complex ($c_{\mathrm{hybrid}}>6$) tiers, and assigned to three progressive stages with increasing data complexity and corresponding training configurations. In addition to gradually expanding the training set from simple to more challenging samples, the three stages also adjust the detector input resolution, optimization parameters, loss weighting, and augmentation strength to match the increasing difficulty of the learning task.

The values 512, 640, and 768 denote the detector input resolution used in the YOLO training framework. Although all preprocessed CT slices were saved at a spatial resolution of 512×512, the images were internally resized to the specified detector input resolution before feature extraction and prediction. Accordingly, Stage 1 uses the original image scale, whereas Stage 2 and Stage 3 resample the same CT slices to 640×640 and 768×768, respectively. This progressive increase in detector input resolution is designed to align with the curriculum learning strategy. In the early stage, a lower input resolution provides a simpler optimization setting, allowing the model to first learn stable and coarse lesion representations from relatively simple samples. In later stages, higher input resolutions preserve more fine-grained structural information and may therefore facilitate the detection of subtle nodules and more accurate localization in more challenging cases.

Stage 1 (512 px)Author: the bold format has been removed.

Training was initialized using simple labeled samples together with high-quality negative samples for 50 epochs. The learning rate was set to $\eta_{1}=0.003$, with loss weights of box = 2.0, cls = 4.0, and dfl = 0.1. Data augmentation included 3° rotation, 5% translation, and 10% scaling.

Stage 2 (640 px) Training was then expanded to include both simple and medium labeled samples, along with high- and medium-quality negative samples, for 100 epochs. The learning rate was reduced to $\eta_{2}=0.002$, and the loss weights were adjusted to box = 5.0, cls = 2.0, and dfl = 0.5. Augmentation strength was increased to 8° rotation, 10% translation, and 20% scaling.

Stage 3 (768 px) In the final stage, the full training set was used, including all complexity levels and all negative samples, for 100 epochs. The learning rate was further decreased to $\eta_{3}=0.001$, with loss weights of box = 7.0, cls = 1.5, and dfl = 1.0. Stronger augmentation was applied, including 12° rotation, 15% translation, and 30% scaling.

### 3.4. Baseline Training Without Curriculum Learning

The conventional training without curriculum learning (No-CL) setting serves as the baseline and follows the conventional single-stage training paradigm widely adopted in object detection, where all training samples are uniformly shuffled and optimized jointly without curriculum scheduling. Specifically, the training used the Ultralytics framework with a fixed input resolution of 640, batch size 32, and a maximum of 500 training epochs. Optimization is performed using SGD with an initial learning rate of $1\times 10^{-4}$, momentum 0.937, and weight decay $5\times 10^{-4}$, together with early stopping based on validation performance (patience = 30 epochs). The loss configuration is fixed throughout training (box = 5.0, cls = 2.0, dfl = 0.3), and evaluation uses a confidence threshold of 0.1 and an IoU threshold of 0.5.

### 3.5. Experimental Implementation

All experiments were implemented using the YOLOv11 framework, which serves as a strong baseline for lung nodule detection in recent studies [29]. Model training was conducted on NVIDIA A100 GPUs with automatic mixed precision enabled to improve computational efficiency and reduce memory consumption. All major training hyperparameters were determined through validation-based grid search and empirical tuning, while keeping the test set untouched. The search covered key settings including input resolution, learning rate, loss weights, and augmentation-related parameters. Final configurations were selected primarily based on validation ${\mathrm{mAP}}_{50}$, with additional consideration of training stability and overall detection performance. For the hybrid curriculum framework, stage-specific hyperparameters were chosen to support stable optimization as training progressed from easier to harder samples.

## 4. Results

### 4.1. Evaluation Metrics

Detection performance was evaluated using standard object detection metrics, including ${\mathrm{mAP}}_{50}$, ${\mathrm{mAP}}_{50–95}$, precision, and recall. For a given intersection-over-union (IoU) threshold τ, a predicted bounding box is considered a true positive (TP) if its IoU with a ground-truth box satisfies IoU≥τ; otherwise, it is counted as a false positive (FP), while unmatched ground-truth boxes are treated as false negatives (FN). Precision and recall are defined as

(4) $$\mathrm{Precision}=\frac{\mathrm{TP}}{\mathrm{TP}+\mathrm{FP}}\;\mathrm{Recall}=\frac{\mathrm{TP}}{\mathrm{TP}+\mathrm{FN}}$$

Average Precision (AP) is defined as the area under the precision–recall curve at a given IoU threshold τ,

(5) $$\mathrm{AP}\left(\tau \right)=\int_{0}^{1}P\left(R,\tau \right)\;dR,$$

where P(R,τ) denotes precision as a function of recall. The primary evaluation metric in this study is ${\mathrm{mAP}}_{50}$, defined as

(6) $${\mathrm{mAP}}_{50}=\mathrm{AP}\left(0.50\right),$$

which emphasizes detection performance under a commonly used IoU criterion and serves as the main indicator for model comparison. In addition, we report ${\mathrm{mAP}}_{50–95}$, computed as

(7) $${\mathrm{mAP}}_{50–95}=\frac{1}{10}\sum_{\tau \in \{0.50,0.55,\ldots ,0.95\}}\mathrm{AP}\left(\tau \right),$$

which provides a stricter assessment of localization quality by averaging performance across multiple IoU thresholds. All model selection and hyperparameter tuning were performed using the validation set. After fixing all training configurations, the selected models were evaluated on the held-out test set, and all results reported in the following sections correspond to test-set performance.

### 4.2. Comparison of Curriculum Learning Strategies

We compare four training strategies: conventional training without curriculum learning (No-CL), manual-only curriculum learning based on handcrafted difficulty, detector-only curriculum learning based on model-driven difficulty, and the proposed hybrid curriculum learning strategy. The No-CL setting serves as the baseline and follows the conventional single-stage training paradigm widely adopted in object detection, where training samples are uniformly shuffled and optimized jointly without explicit difficulty modeling. All methods use the same YOLOv11s detector and share the same dataset split, preprocessing pipeline, and evaluation protocol described in Section 3. The detailed training configurations of the hybrid curriculum and No-CL baseline are provided in Section 3.3 and Section 3.4, respectively.

As shown in Table 1, all curriculum learning strategies outperform the conventional No-CL baseline, demonstrating the effectiveness of difficulty-aware training for lung nodule detection. Compared with standard training without curriculum scheduling, introducing difficulty-aware sample organization consistently improves ${\mathrm{mAP}}_{50}$, ${\mathrm{mAP}}_{50–95}$, recall, and precision, indicating that progressively presenting training samples helps stabilize optimization and improves generalization.

Manual-only curriculum learning and detector-only curriculum learning both achieve clear performance gains over the No-CL baseline, indicating that difficulty-aware data organization improves optimization compared with conventional random training. The proposed hybrid curriculum achieves the best overall performance in terms of ${\mathrm{mAP}}_{50}$, recall, and precision, improving ${\mathrm{mAP}}_{50}$ from 0.672 to 0.696 and recall from 0.588 to 0.634. In addition, statistical analysis showed that the improvement of Hybrid CL over No-CL in the primary metric ${\mathrm{mAP}}_{50}$ was statistically significant (p<0.05). These results suggest that combining handcrafted priors with optimization-aware difficulty signals provides more effective curriculum guidance than relying on either source alone.

Although the manual-only curriculum achieves a slightly higher ${\mathrm{mAP}}_{50–95}$, the hybrid strategy demonstrates a more favorable balance between detection sensitivity and precision, indicating stronger overall detection behavior while maintaining competitive localization quality.

**Figure 3:**
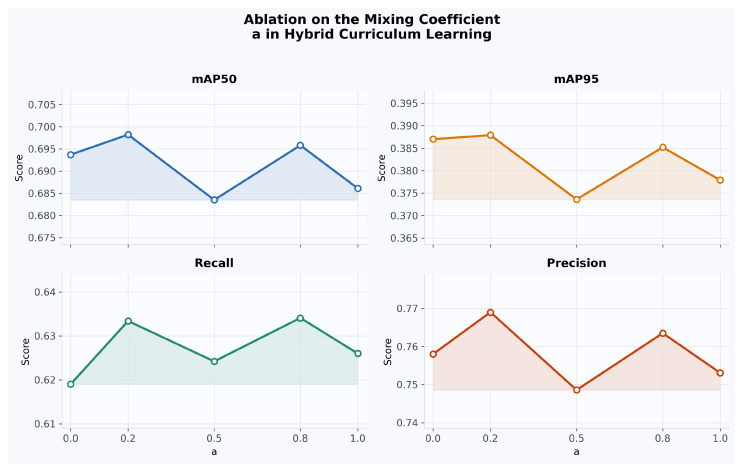
Ablation study on the mixing coefficient *a* in the hybrid curriculum score.

To further examine the respective roles of handcrafted and detector-driven difficulty, we conducted an ablation study on the mixing coefficient *a*, which controls their relative contribution in the hybrid curriculum score. Here, a=0 corresponds to a purely detector-driven curriculum, while a=1 corresponds to a purely handcrafted curriculum. As shown in Figure 3, the best ${\mathrm{mAP}}_{50}$ is achieved at an intermediate value, specifically a=0.2, rather than at either extreme. This result further supports that handcrafted and detector-driven difficulty signals provide complementary rather than redundant information for curriculum construction.

Overall, the results indicate that handcrafted and detector-driven difficulty signals provide complementary information. By integrating both into a unified curriculum, the hybrid strategy yields more stable optimization and consistently improved detection performance compared with individual curriculum designs.

### 4.3. Data Efficiency Analysis

To further evaluate the practical benefit of curriculum learning in data-limited scenarios, we conduct additional experiments under reduced training set sizes. This analysis is motivated by the high annotation cost in medical imaging, where detection models are often trained with limited labeled data and can be more susceptible to unstable optimization and overfitting. We therefore assess whether the proposed hybrid curriculum learning strategy improves data efficiency and maintains robust performance when the amount of training data is constrained.

We consider two complementary settings, both constructed at the patient level to prevent information leakage so that all slices from a selected patient are included together and no patient appears across multiple subsets. (1) Proportional reduction: we subsample the original training set to retain {1/2, 1/5, 1/10} of the training data. (2) Fixed-size subsets: we construct training subsets with fixed numbers of slices (5000, 10,000, 20,000, and 40,000 slices), where patients are sequentially included until the target number of slices is reached. In both settings, the validation and test sets remain unchanged, and all evaluation results are reported on the held-out test set.

Table 2 summarizes the detection performance under proportional reduction of the training data. For each fraction, we perform *n*-fold patient-level subsampling and report the average performance on the held-out test set, which reduces the sensitivity to a particular subset selection. Overall, Hybrid CL consistently outperforms the No-CL baseline across all fractions in terms of ${\mathrm{mAP}}_{50}$ and ${\mathrm{mAP}}_{50–95}$, demonstrating improved data efficiency under limited supervision. In particular, when using 1/2 of the training data, Hybrid CL improves ${\mathrm{mAP}}_{50}$ from 0.642 to 0.673 and ${\mathrm{mAP}}_{50–95}$ from 0.351 to 0.387, accompanied by a recall gain from 0.556 to 0.586. Under a more constrained regime (1/5), Hybrid CL maintains consistent gains across all four metrics, indicating that difficulty-aware sample scheduling remains beneficial even when the training set is substantially reduced.

When the training data is reduced to 1/10, Hybrid CL still improves ${\mathrm{mAP}}_{50}$ from 0.532 to 0.541 and ${\mathrm{mAP}}_{50–95}$ from 0.269 to 0.297, and yields a notable precision gain from 0.589 to 0.629, while recall slightly decreases from 0.463 to 0.449. This trend suggests that, under extremely limited training data, the hybrid curriculum may prioritize more conservative predictions that reduce false positives, thereby improving precision, but may also suppress some borderline true positives, leading to a modest recall drop.

We further evaluate fixed-size training subsets with fixed numbers of slices (5000, 10,000, 20,000, and 40,000). All fixed-size subsets are generated using patient-level sampling, with a fixed random seed (seed = 42). As shown in Table 3, Hybrid CL consistently improves ${\mathrm{mAP}}_{50}$ across all training set sizes, with the improvement becoming more apparent as the amount of training data increases. For small subset sizes (5000 and 10,000 slices), Hybrid CL yields modest improvements in ${\mathrm{mAP}}_{50}$ from 0.488 to 0.494 and from 0.543 to 0.554, respectively, and substantially increases precision from 0.615 to 0.654 and from 0.632 to 0.705, while recall decreases from 0.331 to 0.306 and from 0.418 to 0.374. This again suggests that, under very limited training data, the hybrid curriculum tends to produce a more conservative detector, improving precision but potentially missing some true nodules.

With larger training set sizes (20,000 and 40,000 slices), Hybrid CL improves both detection accuracy and sensitivity. At 20,000 slices, Hybrid CL improves ${\mathrm{mAP}}_{50}$ from 0.604 to 0.635 and recall from 0.516 to 0.564, while maintaining comparable precision, which increases slightly from 0.648 to 0.650. At 40,000 slices, Hybrid CL further improves ${\mathrm{mAP}}_{50}$ from 0.688 to 0.715, ${\mathrm{mAP}}_{50–95}$ from 0.398 to 0.420, recall from 0.604 to 0.612, and precision from 0.703 to 0.764. These results indicate that the proposed curriculum provides more consistent and balanced improvements when a moderate amount of training data is available.

An additional observation is that training on a fixed subset of 40,000 slices achieves stronger performance than the full-data setting reported earlier. For example, the ${\mathrm{mAP}}_{50}$ improves from 0.696 to 0.715. We hypothesize that this may be due to the presence of a small portion of samples in the full dataset that negatively affect generalization, such as low-quality slices, ambiguous negatives, or noisy and approximate annotations that introduce optimization noise. Subsampling may implicitly exclude some of these detrimental cases, resulting in a cleaner effective training distribution and improved test performance. Another plausible factor is stochasticity in subset construction and optimization. Since the 40,000-slice result is obtained from a single sampling seed, the observed improvement may partially reflect a favorable subset composition.

Taking together, both proportional reduction and fixed-size subset experiments support the conclusion that Hybrid CL improves data efficiency compared with conventional training, with particularly strong and balanced gains emerging at moderate training budgets. Under extremely limited data, Hybrid CL tends to yield higher precision but may reduce recall, suggesting a conservative prediction regime. These findings emphasize the practical importance of difficulty-aware curriculum learning in medical imaging scenarios where annotated data are expensive and limited, and further motivate future investigations into curriculum design and decision threshold calibration to achieve better precision-recall balance under low-data regimes.

### 4.4. Case Study

**Figure 4:**
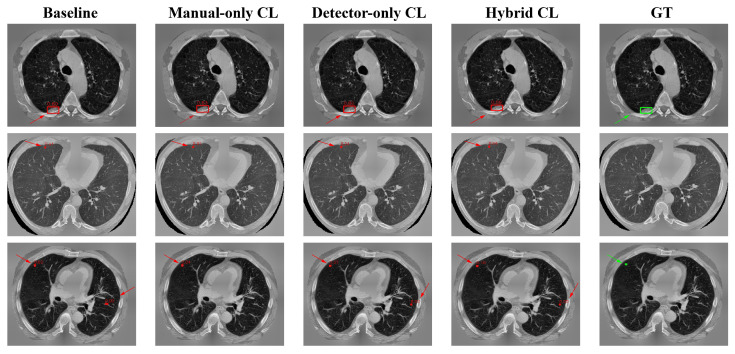
Qualitative comparison of detection results on representative LUNA25 test slices. From left to right: Baseline (No-CL), Manual-only CL, Detector-only CL, Hybrid CL, and ground truth (GT). Red boxes denote predicted bounding boxes and green boxes indicate ground-truth annotations. Confidence scores are shown above each predicted box.

To qualitatively analyze the effect of different curriculum learning strategies, we present representative detection examples from the LUNA25 test set in Figure 4. The figure compares predictions produced by the baseline model without CL, manual-only CL, detector-only CL, and the proposed hybrid CL, together with the ground-truth annotations.

As shown in Figure 4, the first row presents a lesion attached to the chest wall, and all four models successfully detect the nodule. The hybrid CL model produces the highest confidence score of 0.89, while the other methods yield comparatively lower confidence values for the same lesion. In the second row, all models generate false-positive detections in vessel-like structures. Although none of the approaches completely avoids incorrect responses, the hybrid CL model assigns a confidence score of 0.04 to the false-positive prediction, which is lower than those produced by the other models. The third row corresponds to a very small pulmonary nodule. In this case, the hybrid CL model reports a detection confidence of 0.16, whereas the confidence scores generated by the remaining models are all below 0.1. Together, these examples illustrate the differences in detection confidence and prediction behavior across the evaluated CL strategies.

## 5. Discussion

This study provides a systematic investigation of CL for CT-based lung nodule detection and demonstrates that difficulty-aware training can consistently improve detection performance and data efficiency on the LUNA25 benchmark. By integrating handcrafted and model-driven difficulty signals into a unified hybrid framework, we establish a structured optimization paradigm that departs from conventional random sample scheduling. The results suggest that explicitly modeling sample difficulty is not merely a training heuristic, but a practical mechanism for improving robustness and generalization in heterogeneous medical imaging datasets. In this sense, our work offers both an empirical benchmark and a methodological reference for future detection-oriented CL research.

Despite these findings, several aspects of CL design remain open for further refinement. The current framework adopts a predefined three-stage schedule with fixed hyperparameters and difficulty thresholds. While this design ensures interpretability and reproducibility, it does not dynamically adapt to the model’s evolving learning state. In addition, the handcrafted coefficients and thresholds, as well as the model-driven weights used in this study, are heuristic and dataset-dependent rather than universally optimal. More advanced adaptive CL strategies such as uncertainty-aware sampling, confidence calibrated reweighting, or dynamic curriculum transitions, may further enhance flexibility and optimization efficiency. Additionally, automated tuning of the mixing coefficient between handcrafted and detector-driven difficulty could improve portability across datasets and model architectures. It is also worth noting that the slice sampling strategy retains all nodule slices while subsampling background slices, shifting the nodule-to-background ratio away from its natural distribution. This may affect model calibration and evaluation reliability in real-world deployment, and future work should assess detection performance under more realistic slice composition settings.

Another important consideration is the dimensionality of detection. The present study focuses on slice-wise 2D detection for computational efficiency and compatibility with widely adopted object detection frameworks. However, pulmonary nodules are inherently three-dimensional structures with volumetric continuity. Extending CL-based training strategies to 3D or hybrid 2.5D architectures would provide a more comprehensive validation and may unlock additional performance gains by incorporating volumetric context into difficulty estimation. Future work may also benefit from integrating radiomic signatures, which have shown value in pulmonary nodule characterization [41], as complementary information for richer lesion representation and improved clinical relevance.

Although LUNA25 is currently one of the largest publicly available lung nodule datasets with enhanced annotation quality, it remains a curated benchmark dataset. Imaging protocols, scanner vendors, and patient populations may not fully capture real-world variability. Therefore, evaluating CL-based optimization strategies on multi-center datasets with broader distributional diversity will be critical for assessing robustness under domain shift. Such validation would further clarify the generalizability of difficulty-aware training in practical deployment settings.

Finally, while quantitative detection improvements are encouraging, translation into clinical practice requires further validation. Beyond mAP and recall, future studies should examine clinical endpoints such as reduction of false-positive burden, impact on radiologist reading time, integration with malignancy risk stratification, and longitudinal monitoring workflows. Prospective studies and human-in-the-loop evaluation will be essential to determine the real-world clinical utility of structured CL-based optimization.

Overall, this work highlights the importance of structured difficulty-aware training in medical object detection and demonstrates that CL can serve as an effective optimization strategy beyond architectural modifications. By establishing a reproducible benchmark on LUNA25 and introducing a hybrid difficulty modeling framework, this study lays a methodological foundation for future research on data-efficient and robust detection systems in medical imaging.

## 6. Conclusions

In this work, we presented a systematic study of CL for CT-based lung nodule detection on the enhanced LUNA25 benchmark and introduced a hybrid difficulty modeling strategy that integrates handcrafted priors with detector-driven signals to construct a progressive training curriculum. Across full-data training and multiple reduced-data regimes, the hybrid CL setting consistently improves detection performance over conventional training without CL and over single-source curricula, demonstrating that jointly leveraging intrinsic image complexity and optimization-aware feedback provides effective guidance for sample scheduling. Beyond quantitative gains, our study establishes a reproducible baseline for YOLOv11-based detection on LUNA25 and highlights structured difficulty-aware training as a practical direction for improving robustness and data efficiency in heterogeneous medical imaging datasets. Future work will extend the proposed framework to volumetric 3D detection, validate generalization on broader multi-center cohorts, and conduct further clinical evaluation to assess real-world utility and workflow impact.

## Figures and Tables

**Table 1:** Comparison of different curriculum learning strategies on the LUNA25 test set. All curriculum learning variants outperform the No-CL baseline. The proposed hybrid curriculum achieves the best performance in the primary metric (${\mathrm{mAP}}_{50}$) as well as precision and recall, while maintaining competitive ${\mathrm{mAP}}_{50–95}$ performance.

Method	${\mathrm{mAP}}_{50}$↑	${\mathrm{mAP}}_{50--95}$↑	Recall ↑	Precision ↑
No-CL (Baseline)	0.672±0.003	0.369±0.006	0.588±0.002	0.725±0.009
Manual-only CL	0.694±0.008	0.387±0.001	0.619±0.004	0.758±0.022
Detector-only CL	0.686±0.002	0.378±0.003	0.626±0.002	0.753±0.003
**Hybrid CL (Ours)**	0.696±0.006	0.385±0.003	0.634±0.004	0.764±0.006

**Table 2:** Performance comparison between No-CL and Hybrid CL under proportional reduction of the training data.

Training Fraction	Method	${\mathrm{mAP}}_{50}$↑	${\mathrm{mAP}}_{50–95}$↑	Recall ↑	Precision ↑
1/2	No-CL	0.642	0.351	0.556	0.696
	Hybrid CL (Ours)	0.673	0.387	0.586	0.695
1/5	No-CL	0.574	0.299	0.494	0.644
	Hybrid CL (Ours)	0.599	0.327	0.501	0.659
1/10	No-CL	0.532	0.269	0.463	0.589
	Hybrid CL (Ours)	0.541	0.297	0.449	0.629

**Table 3:** Performance comparison between No-CL and Hybrid CL using fixed-size training subsets. Results are reported on the LUNA25 test set.

Training Budget	Method	${\mathrm{mAP}}_{50}$↑	${\mathrm{mAP}}_{50–95}$↑	Recall ↑	Precision ↑
N = 5000	No-CL	0.488	0.279	0.331	0.615
	Hybrid CL (Ours)	0.494	0.281	0.306	0.654
N = 10,000	No-CL	0.543	0.318	0.418	0.632
	Hybrid CL (Ours)	0.554	0.324	0.374	0.705
N = 20,000	No-CL	0.604	0.345	0.516	0.648
	Hybrid CL (Ours)	0.635	0.366	0.564	0.650
N = 40,000	No-CL	0.688	0.398	0.604	0.703
	Hybrid CL (Ours)	0.715	0.420	0.612	0.764

## Data Availability

The original imaging data are publicly available. The processed dataset and derived annotations generated during this study are available from the corresponding author upon reasonable request.
